# Identiﬁcation and treatment of obstructive sleep apnea by a primary care team with a subset focus on chronic pain management

**DOI:** 10.1371/journal.pone.0237359

**Published:** 2020-08-13

**Authors:** Kathleen Whittington, Leigh Simpson, Michael Clay, Joanna Tierney, Dixie Harris

**Affiliations:** 1 Internal Medicine, Intermountain Healthcare, Sandy, Utah, United States of America; 2 Intermountain Healthcare, Sandy, Utah, United States of America; 3 Pulmonary and Sleep Medicine, Intermountain Healthcare, Sandy, Utah, United States of America; Harper University Hospital, UNITED STATES

## Abstract

**Background:**

Patients diagnosed with obstructive sleep apnea (OSA), who also consume prescription opioids, have a greater likelihood of morbidity and mortality. This study evaluated whether a primary care team, focused on chronic pain care management, could use a validated questionnaire (STOP-Bang) and motivational follow-up, to increase identification and treatment of OSA.

**Methods:**

This study was a retrospective, dual arm, pre/post controlled study. Participants of this study included the complete chronic pain management sub group treated by this primary care team. Participants were ≥ 18 years old and prescribed daily opioids for treatment of chronic pain. All participants had a multifaceted, individualized, educational meeting that included completing a STOP-Bang questionnaire. Participants who received a score ≥ three were advised to follow up with their primary care physician. Participants were seen quarterly throughout the study.

**Results:**

The primary outcome of this study was that 65% of participants with likely OSA were using CPAP for a minimum of 12 months (range of 12–25 months, 18-month average) post-intervention vs. 37% CPAP-use in the control group (12 months of observation), both groups were chronic opioid users with OSA. This was a 28% relative improvement (p = 0.0034). A secondary outcome was that 8.9% of non-prior CPAP users obtained CPAP post- intervention; a 56.7% pre-post improvement (p = 0.0064, x^2^ = 10.08 with 1 degree of freedom). Also, participants who were likely to have OSA (STOP-Bang score ≥ 3 or had a positive polysomnography (AHI >5 with comorbidities)) compared to those unlikely to have OSA (STOP-Bang score <3 or had a negative polysomnography (AHI <5)) in this study were more likely to be male, have a higher BMI, have hypertension, have cardiovascular disease and/or have diabetes (all types).

**Conclusion:**

Team based care management for participants taking prescription opioids, where STOP-Bang questionnaires were completed, were associated with an increase in the identification and treatment of OSA.

## Introduction

### Study rationale

The objective of this study was to evaluate whether a team-based care management intervention would increase identification and treatment of obstructive sleep apnea in participants taking medication for chronic pain.

### Current knowledge

Obstructive sleep apnea (OSA) is a chronic sleep disorder characterized by episodes of apneas and hypopneas, or the complete or partial collapse of the upper airway. [1] A diagnosis is made when a patient has 15 or more of these events in an hour, or five of these events if other predicting symptoms for OSA are present. [2] Approximately 18 million American adults have this sleep disorder. Patients with moderate to severe OSA are often treated with a continuous positive airway pressure device, or CPAP. Treatment can reduce the risks of hypertension, coronary artery disease, heart failure, arrhythmias, sudden cardiac death, and stroke. [2] A validated questionnaire (STOP-Bang) score can be used as a screening tool to identify patients at risk for OSA. A STOP-Bang sleep questionnaire is commonly distributed to patients who show signs of having potential risk factors for OSA. “STOP” questions snoring, tiredness, observed apnea, and high blood pressure, and “Bang,” BMI > 35 kg/m^2^, Age > 50 years, Neck circumference > 40 cm, and Gender (male). [1, 3–6] A score of three or higher on STOP-Bang places patients at higher risk of OSA requiring further evaluation, which includes an overnight polysomnogram, or sleep study, to confirm diagnosis. [1]

Prescription opioid use for pain management is still a common practice in primary care settings, though the rates of overdose due to these drugs in the United States are at an unprecedented high with 70, 237 deaths in 2017. [7–9] Patients using opiates for chronic pain management have also been found to have a greater likelihood of developing central sleep apnea, which worsens the severity of OSA, and ataxic breathing. [10] A relationship exists between pain and sleep disruption with over 70% of patients who have chronic pain having reported difficulty sleeping, which increases hyperalgesia. [11, 12] An analgesic effect may be present, where pain creates sleep disorders and a shortage of sleep increases pain. [11, 12] Patients with OSA who are then prescribed opioids have an increased risk of opioid-induced respiratory depression (OIRD). [13] Despite the potential morbidity of the combination of OSA, central sleep apnea and Opioid use, studies have shown that those that have this combination only remain on definitive treatment for their OSA (CPAP) 37% of the time. [14] It is, therefore, of the utmost importance that patients who chronically use opioids are evaluated for OSA, and those diagnosed, adhere to CPAP.

In order to decrease mortality, it is necessary to educate patients on the risks and health concerns related to taking opioids. Brief interventions can influence behavior of patients who are at high risk for abuse of a substance. [15] Individualized, nurse-led management education is a tool that can be used as a brief intervention within a team-based approach [16] These individualized, information gathering, motivational meetings allow patients to experience empathy as their medical needs are met. [17, 18] Individualized, nurse-led management education is a tool that can be used as a brief intervention within a team-based approach. [16] Instruction and guidance can be coupled with an assessment of a patient's potential risk factors for comorbidities such as OSA using a STOP-Bang questionnaire.

## Materials & methods

### Design

This study was a retrospective, dual arm, pre/post controlled study to evaluate whether an intervention (STOP-Bang questionnaire education and motivational follow-up) would improve OSA diagnosis and treatment compliance. The first investigation (arm) compared the control group (daily opioid use with OSA) to the part of the intervention group with OSA on CPAP or at risk for OSA (STOP-Bang ≥3). This arm’s purpose was to determine whether the intervention increased treatment compliance with CPAP. The second investigation (arm) compared the study group to itself pre/post intervention. This arm evaluated whether applying the intervention increased diagnosis and treatment of OSA. The single independent variable in this study was a participant's admission to the chronic pain management sub-group with the administration of a STOP- Bang questionnaire, and the dependent variable was CPAP use. Inclusion criteria consisted of ongoing participation in primary care pain management sub group, daily opioid use, and an age ≥ 18.

**Fig 1:**
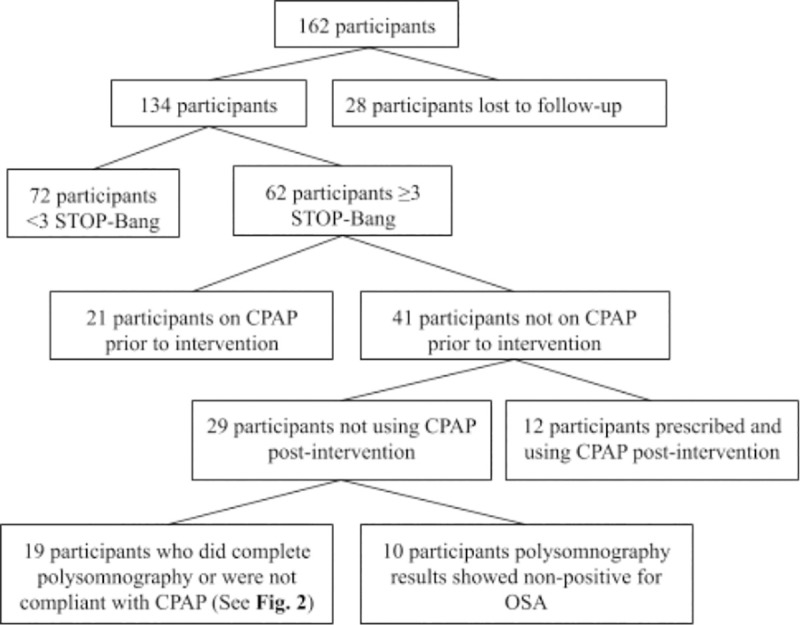
Participant retention.

This study occurred between October 2017 and January 2020 which included 15 months for exposure and then at least 12 months of follow up. One of Intermountain Healthcare's outpatient facilities in Utah served as the site for this study. The total number of patients during the duration of the study who entered into the pain management sub group, ≥ 18 years old, and were taking a daily dose of opioids, was 162. Out of the 162 identified participants, 134 participants remained in the clinic during the entirety of this study. (Fig 1) Ethics approval (IRB Number: 1050773) for this study was obtained from the Institutional Review Board of Intermountain Healthcare. Consent was waived for this retrospective study.

### Controls

A Medline and Pubmed basic search from 2014–2019 was used to identify a historical yet contemporary United States based control group that had Obstructive Sleep Apnea and met the inclusion criteria of being ≥ 18 years old, and were taking a daily dose of opioids. The control that was chosen was comparable to this study’s intervention group with regard to Race, Age, Gender, Morphine Equivalent Daily Dose and Comorbidities. Also, the control used came from a study that lasted a similar duration. [14]

### Subjects

**Fig 2:**
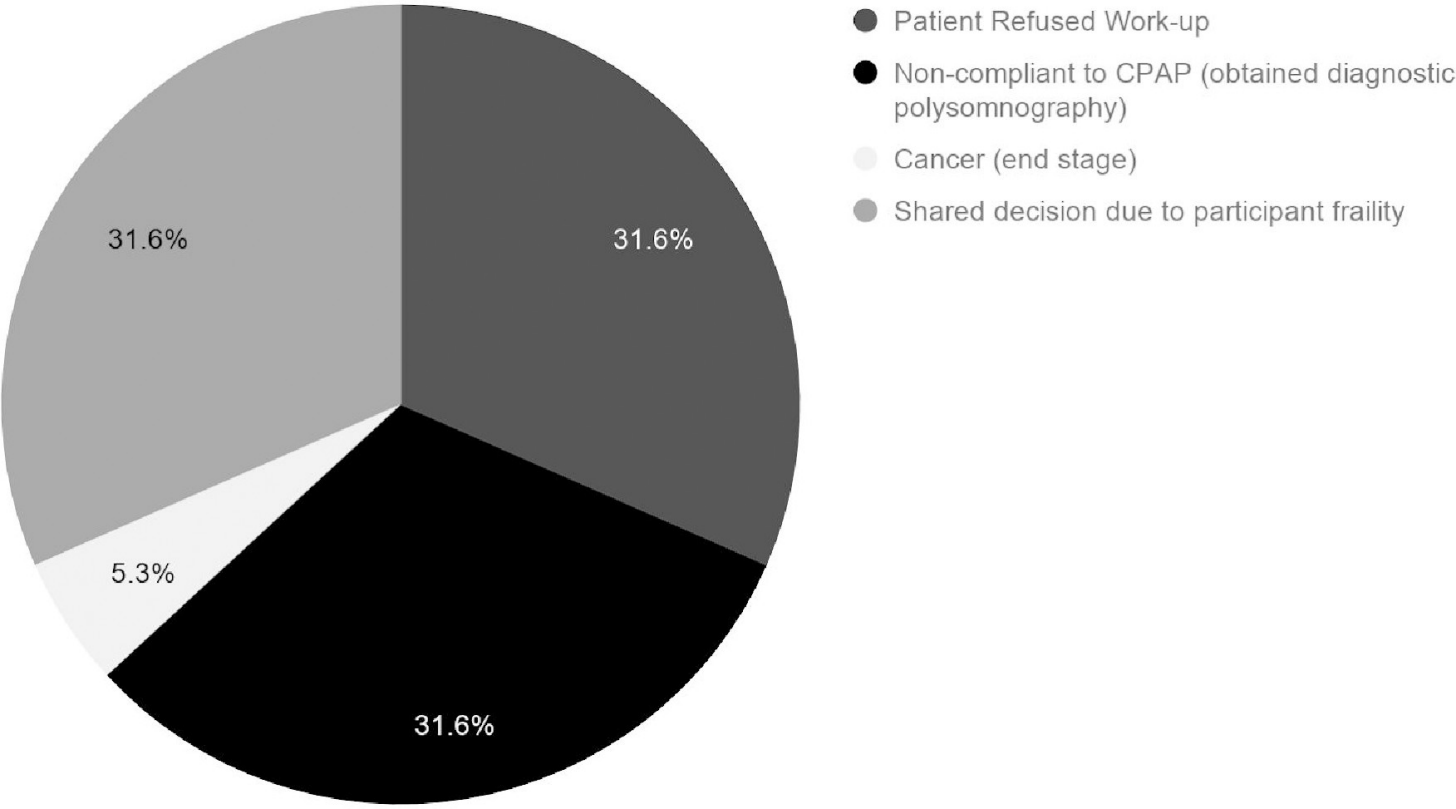
19 participants at high risk for sleep apnea (STOP-Bang score ≥ 3) who did not undergo diagnostic evaluation or complete treatment.

Each participant received an information gathering and educational session with a registered nurse in the primary care facility at the time of their admittance into the chronic pain management program between 2017–2020. During this session, a full pain history was obtained from participants. All participants in the study underwent STOP-Bang screening. All participants who were at high risk for OSA (STOP-Bang ≥ 3) were referred back to their primary care physician. Participants and physicians made joint decisions during a follow-up appointment whether to pursue polysomnography. Reasons why participants did not complete polysomnography or attain treatment for OSA are that the participants refused work-up, there was a shared decision due to participant frailty, had cancer (end stage), or were non-compliant to CPAP. (Fig 2)

Each participant received initial in-person education with the same registered nurse regarding non-pharmacological treatment of pain, self-help groups, risks and benefits of treatment of pain management, compliance with treatment of pain and its comorbidities, and information about Naloxone (an opioid reversal agent). This education was provided in a 1 hour session using the FRAMES (Feedback, Responsibility, Advise, Menu for Change, Empathy and Enhancing Self Efficacy) motivational method. [19] A chronic pain management agreement was executed. Additional information obtained during this session included a Current Opioid Misuse Measure and a STOP-Bang questionnaire. All participants who received a score greater than or equal to three on the STOP-Bang were told to follow up with their primary care physician. All primary care providers were made aware they would receive electronic data on STOP-Bang questionnaires and participants with scores ≥ 3 would make a follow up appointment with them. Upon completion of this session information was recorded electronically and transmitted to the primary care physician on record. Each participant also had quarterly, in-person, follow-up appointments with their primary care physician. In addition, quarterly, in-person or phone visits, with the pain management nurse were continued. Physician visits varied in length and scope. Individualized nursing visits, that continued to use the FRAMES method and lasted for a duration of 20 minutes, were focused on remaining compliant with all ongoing therapies. The nurse would document the interaction and any concerns about compliance with any therapy in the patient's EMR (Electronic Medical Record) at the conclusion of the visits. The information was based on the subjective input received from the participant. Quarterly follow-up remained consistent throughout the study. Attending all follow-up appointments, both with the primary care physician and the pain management nurse, was a prerequisite to remain in the pain management group. Remaining in the pain management was group was the only avenue to receive controlled pain medication at this facility.

### Data collection

The baseline biometric parameters of Race, Gender, BMI, Age, Tobacco Use, Alcohol Use, and Morphine Equivalence Daily Dosage (MEDD) were extracted from each participant’s electronic medical record (EMR). Collected data also included information on Chronic Disease (Hypertension, Cardiovascular Disease, Pulmonary Disease, Thyroid Disease, Diabetes, Gastroesophageal Reflux Disease, and Chronic Kidney Disease) and specific medication types (Benzodiazepines and Pregabalin). STOP-Bang scores were gathered during the initial chronic pain management encounter when participants joined the study. Subjective data on CPAP adherence was found in the EMR documentation of the pain management nurse and primary care physician follow up reports (post-inclusion in the study).

### Statistical methods

Means and standard deviations were calculated for demographic variables. The D-Agostino-Pearson test was used to confirm normality. [20] Unequal variance t-testing was used in the statistical analysis of both arms of this study to compare Age, BMI, and Morphine Equivalent Daily Dosing. Two proportion Z-testing was used to compare the control group (daily opioid use with OSA) to the participants in the intervention group with OSA (on CPAP) or at high risk for OSA (STOP-Bang ≥ 3) with regards to CPAP use, Race, Gender, Diabetes, Cardiovascular Disease, and Hypertension. Two proportion Z-testing was also used to compare the participants not likely to have OSA (STOP-Bang <3) or had a negative polysomnography, to those with OSA (on CPAP) or at high risk for OSA (STOP-Bang ≥ 3, not completing polysomnography or not compliant with CPAP). A 2 x 2 contingency table using McNemar’s test, with correction for continuity, was used to compare the participants’ CPAP use pre/post intervention. [21]

### IRB approval

IRB Approval was granted from Intermountain Healthcare IRB (IRB Number: 1050773).

## Results

**Fig 3:**
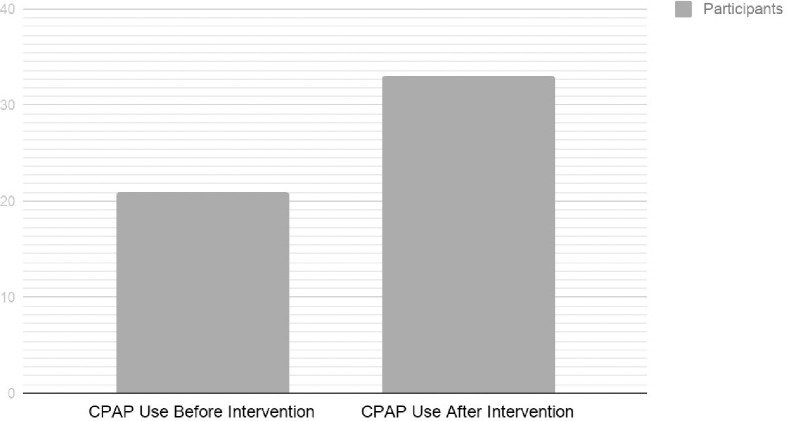
CPAP use before and after intervention.

The primary outcome of this study was that 65% of participants with likely OSA were using CPAP for a minimum of 12 months (range of 12–25 months, 18-month average) post-intervention vs. 37% CPAP use in the control group (12 months of observation), which were chronic opioid users with OSA. This was a 28% relative improvement (p = 0.0034). A secondary outcome was that 8.9% of non-prior CPAP users obtained CPAP post intervention; a 56.7% pre-post improvement (p = 0.0064, x^2^ = 10.08 with 1 degree of freedom). (Fig 3) This resulted in 24.6% of intervention participants being treated with CPAP. Comparing the average number of intervention participants with OSA (on CPAP) or at risk for OSA (STOP-Bang ≥ 3) to the control data set, resulted in significant differences for BMI (<0.0001) and Male Gender (<0.0001). Other demographics between these two groups; Age (p = 0.074), MEDD (p = 0.98), Race (Caucasian, p = 0.1), Hypertension (p = 0.16), Cardiovascular Disease (p = 0.19) and Diabetes (all forms, p = 0.53), showed no statistically significant differences between groups. Comparing the average number of intervention participants with OSA (on CPAP) or at risk for OSA (STOP-Bang ≥ 3) to the average number of intervention participants not likely to have OSA (STOP-Bang < 3) or had a negative polysomnography, resulted in the following statistically significant differences: BMI (p = 0.0017), Male Gender (p<0.0001), Hypertension (p = 0.0014), Cardiovascular Disease (p = 0.020) and Diabetes (all forms, p = 0.0009). Other demographics between these two groups; Age (p = 0.48), MEDD (p = 0.94), Race (Caucasian, p = 0.27), Benzodiazepine Use (p = 0.31), Pregabalin Use (p = 0.87) and Active Chronic Disease States: Gastroesophageal Reflux (p = 0.089), Chronic Kidney Disease (0.57), Thyroid Disease (p = 0.83), and Pulmonary Disease (p = 0.66), showed no statistically significant differences between groups. (Table 1)

**Table 1:** Comparison of clinical characteristics (S1 Appendix).

Demographics	Average Number of Participants with OSA (on CPAP) or at risk for OSA (STOP-Bang ≥ 3)	Control (Daily Opioid Use with OSA)	P-value	Average Number of Participants Not Likely to Have OSA (STOP-Bang < 3) or Neg- Polysomnography	Pre-Post P-value
Age	60.5 +/- 11.6	57.2 +/- 10.1	0.074	61.5 +/- 15.4	0.48
BMI ***	31.8 +/- 6.8	35.1 +/- 6.8	<0.0001	28.7 +/- 6.3	0.0017
Morphine Equivalent mg/d at Conclusion of Study ≥ 12 months	33.7 +/- 23.5	33.8 +/- 25.5	0.98	34.0 +/- 23.5	0.94
Demographics	Average Percentage of Participants with OSA (on CPAP) or at risk for OSA (STOP-Bang > 3)	Control (Daily Opioid Use with OSA)	P-value	Average Percentage of Participants Not Likely to Have OSA (STOP-Bang < 3) or Neg- Polysomnography	Pre-Post P-Value
Race (Caucasian)	98%	92%	0.1	100%	0.27
Gender (Male)**	50%	94%	<0.0001	18%	<0.0001
Diabetes***	40%	35%	0.53	11%	0.0009
Cardiovascular Disease**	27%	18%	0.19	11%	0.020
Hypertension*	86%	79%	0.16	56%	0.0014
Gastroesophageal Reflux Disease	15%	N/A	N/A	11%	0.089
Thyroid Disease (All Forms)	29%	N/A	N/A	27%	0.83
Pulmonary Disease (All Varieties)	27%	N/A	N/A	23%	0.66
Active Cancer	9.6%	N/A	N/A	9.8%	0.69
Chronic Kidney Disease	15%	N/A	N/A	11%	0.57
Concurrent Benzodiazepine Use	60%	N/A	N/A	70%	0.31
Concurrent Pregabalin Use	29%	N/A	N/A	28%	0.87

* = p value of < 0.01

** = p value of < 0.05

*** = p value of < 0.001

## Discussion

### Main findings

Current high death rates, compared to healthy subjects, of OSA and chronic pain management with daily opioid use warrant efforts to improve care. [22–24] This study’s findings can be an important step towards improving mortality and morbidity when these two diseases are combined. The results suggested that using a STOP-Bang questionnaire accompanied with motivational education initially, as well as in follow-up, improved diagnosis and treatment of OSA. These findings suggest that team-based care can positively impact the outcome of patients with OSA when they are concurrently on chronic daily opioids and CPAP. These findings warrant additional research. Diagnosed sleep apnea participants who were using CPAP had a significantly higher rate of being male, having hypertension, having cardiovascular disease, having diabetes (all types), and having a higher BMI. The fact that 14.2% of participants at risk for sleep apnea (STOP-Bang ≥ 3) did not receive a full evaluation or end up on definitive treatment highlights the need for continued effort in this field.

### Limitations

The data found in this study regarding CPAP adherence was subject to limitations as well. Some participants that joined this team-based care management approach to treat chronic pain had already been prescribed CPAP and their adherence to the treatment was included in this studies data set, possibly overestimating the conclusions. Adherence to CPAP in this study was based on subjective treatment reconciliation at follow-up appointments with physicians and nurses, and since many participants received their sleep treatment from outside sources, CPAP adherence data, other than confirmation from the patient, was not available. If a participant indicated that they had not been using their CPAP ≥ 50% of the time between any follow-up appointments after the initiation of treatment, they were considered to be non-adherent. This type of data collection is subject to significant recall bias and could potentially inflate the percentage of participant adherence.

This preliminary research was done in the format of a pre/post, dual arm, retrospective study. This type of study has inherent and significant limitations. First, due to the nature of the intervention, team members (nurses and physicians) were not blinded, and continued to evaluate participants throughout the course of this study and could have recommended evaluation and therapy of sleep apnea for alternative reasons. This study could have been affected by a herd mentality, since the primary care physicians worked together in one group and their composite style could have increased or decreased the identification and treatment of sleep apnea. These limitations could be mitigated by performing a large, prospective, multi-center study.

This study is also limited due to the nature of using a historical control. [14] Although the control used mimicked the experimental group in most ways (daily opioid use, MEDD, race, age, and percentages of diabetes, hypertension, and cardiovascular disease), it varied in both BMI (control group had a higher average BMI), and gender (control group had a greater number of male participants). It has been previously demonstrated that a higher BMI favors adherence to CPAP. [25, 26] This does not detract from the finding that the intervention group in this study, which had a lower BMI, had a higher adherence to CPAP. Gender has also been evaluated in previous CPAP compliance studies. Two studies have shown no difference between genders, whereas a different study showed an increase in male non-compliance, and another where there was an increase in male compliance. [26–29] As there is no definitive determination from these studies, it is possible that the variance between the intervention and control groups were due to the gender difference. It would seem from the above studies that there was no strong trend resulting from gender, making gender unlikely to be the cause of the preponderance of the variance.

There was a loss of follow-up with primary care physicians after the administration of the STOP-Bang questionnaire in this study. (Fig 1) Patients may have forgotten to, or chosen not to, schedule a follow-up appointment with their primary care physician after being determined high risk for sleep apnea. The participant received education on six other aspects of opioid managed pain during the one hour informational meeting. Another limitation was that the data from the intervention session was transmitted en-block to the primary care physician electronically. These limitations could have been improved by having the nurse set up a specific appointment to consider sleep disorders in the high-risk group and to either verbally or electronically deliver this information in an isolated approach.

### Comparisons

This study reaffirmed previous studies that showed STOP-Bang was a tool that could identify OSA. [1, 3–6] Other similarities to previous investigations were found. In those studies where daily opioid dosing for pain management was present, 50–71% of patients were found to suffer from OSA and 37% of patients were found to be compliant to CPAP. [14, 30] The data range for previous studies in regard to OSA incidence is consistent with this study in which 61.7% of participants were either diagnosed with OSA on CPAP or were at high risk for OSA (STOP-Bang ≥ 3 not completing polysomnography or not compliant with CPAP). This study was significantly higher with regards to the percentage of remaining on CPAP (65%) suggesting that diligence in repetitive diagnosis attempts and continued follow with motivational techniques may improve care in this type of patient. Also, this study, like others, suggested (p < 0.05) that hypertension, cardiovascular disease, diabetes, male gender, and higher BMI are more common in patients with OSA. [2]

## Conclusions

Left untreated, sleep apnea has a high rate of morbidity and mortality. The use of prescribed opioids, as a form of chronic pain management in a primary care setting, can increase a patient's likelihood of having sleep apnea. A team-based intervention, that included the administration of a STOP-Bang questionnaire, was associated with an increased diagnosis of OSA (56.7% pre-post improvement level). It was also associated with an increased adherence to CPAP (28% relative improvement). The primary reasons CPAP treatment was not received included refusal of evaluation, non-adherence to CPAP, or shared physician/patient decision based on age/frailty.

## Supporting information

S1 Appendix Raw study data set. (XLSX)
